# Comparison of Different Double-Stent Implantation Techniques on Coronary Bifurcation Lesions: A Finite Element Analysis

**DOI:** 10.2174/011573403X404659250702080410

**Published:** 2025-07-11

**Authors:** Hongshuai Cao, Heng Wu, Jingang Zheng, Jingyi Ren

**Affiliations:** 1 Heart Failure Center, Department of Cardiology, China-Japan Friendship Hospital, Beijing, China;; 2 Department of Cardiology, The Central Hospital of Enshi Tujia and Miao Autonomous Prefecture, Enshi, China

**Keywords:** Coronary bifurcation disease, biomechanics, culotte, finite element analysis, crush procedure, IVUS validation

## Abstract

**Introduction:**

This study aims to investigate the impact of different double-stent methods on the structure and mechanics of coronary bifurcation lesions, providing reference indicators for clinicians in selecting an appropriate interventional procedure.

**Methods:**

Three-dimensional reconstruction of coronary Computed Tomography Angiography (CTA) image data of a patient with coronary bifurcation disease was performed. Two types of double-stent (Cullotte and Crush) procedures were simulated, and their effects were evaluated using Finite element analysis. Intravascular Ultrasound (IVUS) validation and retrospective clinical analysis were performed to support computational findings.

**Results:**

The stress distribution following the Cullotte stent was concentrated in the SB, whereas the stress after the Crush procedure was localized at the overlap with the proximal main vessel three-layer stent. Compared with the Crush procedure, the Culotte approach resulted in a lower percentage of double-stent malapposition, better dilation of vascular stenosis, and less narrowing of the SB stent, suggesting a more favorable clinical outcome. IVUS validation and retrospective clinical analysis were performed to support computational findings.

**Discussion:**

Culotte stenting resulted in better stent-vessel conformity and more favorable stress distribution. The findings support FEA as a valuable tool in procedural planning.

**Conclusion:**

The findings suggest that the Culotte technique may offer mechanical advantages over the Crush technique, potentially improving long-term clinical outcomes. These results emphasize the role of computational modeling in optimizing interventional strategies.

## 1 INTRODUCTION

Coronary Bifurcation Lesions (CBLs) are characterized by complex anatomical structure, variable morphology, low immediate surgical success rates, and high risk of long-term cardiovascular events [1, 2]. According to the latest clinical guidelines [3], double-stent implantation becomes essential for bifurcation lesions. Currently, Crush and Culotte are the two commonly used double-stent methods in clinical practice [4].

The evaluation of the double-stent technique for bifurcation lesions has mainly focused on clinical follow-up outcomes. Nonetheless, the conclusions drawn remain ambiguous and inconsistent [5-8]. This may be attributed to a lack of attention to individual variations in coronary artery anatomy among the enrolled patients, and, more importantly, an insufficient consideration of the mechanical injury induced by stent implantation in vessels. It is imperative to investigate double-stent strategies for bifurcation lesions from the perspective of mechanical damage to provide clinicians with comprehensive guidance on optimal choices. However, current clinical technology does not facilitate a quantitative analysis of the mechanical damage to the vascular wall caused by stent expansion during the procedure.

Finite Element Analysis (FEA) is an ideal method for comparing the mechanical behavior of different scaffolds [9]. Published studies have demonstrated the feasibility of using FEA to evaluate interventional therapies [10]; however, to the best of our knowledge, the numerical simulation of coronary bifurcations has not garnered significant attention, particularly from the perspective of different stent implantation methods, although structural mechanics and hemodynamic computational simulations have offered the ability to evaluate quantitative insights [11].

Our team’s previous research employed numerical simulation methods to assess the single stent placement in the Main Vessel (MV) of a coronary bifurcation in real-time, quantitatively evaluating the mechanical damage to diseased vessels caused by different expansion strategies. This study also quantitatively analyzed stent adhesion *via* the morphological parameters of the stent and vessels. Finally, the optimal position of the balloon post-expansion was proposed based on the vascular injury caused by single stent insertion for a bifurcation lesion [12, 13]. These results provided crucial guidance for clinicians in selecting an appropriate post-stent expansion strategy during placement. The aim of this study was to compare two double-stent strategies for bifurcated lesions to provide a scientific basis for optimal selection of the technique.

## 2 MATERIALS AND METHODS

### 2.1 Study Design

This study was designed as a two-stage investigation combining computational modeling with retrospective clinical validation. Initially, FEA was performed to compare the mechanical performance of the Culotte and Crush stenting techniques in a single patient with coronary bifurcation lesions. The simulation results were validated using Intravascular Ultrasound (IVUS) imaging from the same patient. Based on this individualized FEA platform, the methodology was further applied to a retrospective clinical cohort of 50 patients from the China-Japan Friendship Hospital between January 2022 and December 2023 to assess the mechanical performance of the two techniques and validate the feasibility and applicability of FEA in a broader clinical context.

### 2.2 Coronary Stenosis Modeling

**Fig. (1):**
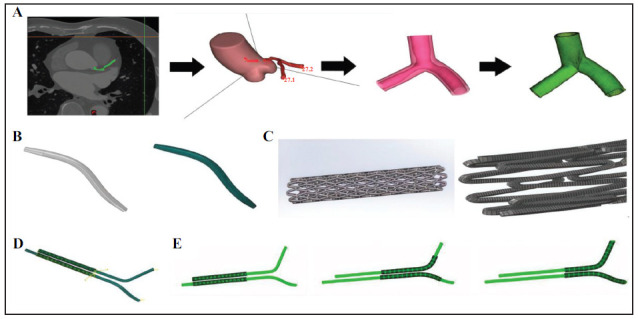
Modeling process, grid division, and support conveying process of coronary artery stenosis. **A.** The patient's CTA data was used to reconstruct the coronary arteries in 3D. The segments E1 and E2 required for the study were intercepted, resulting in a pink 3D coronary model. The model was then meshed (green) to prepare for subsequent calculations; **B.** A 3D model of the balloon, which was then meshed; **C.** A 3D model of the bracket was drawn and then meshed; **D.** The simulated vessels and stent were assembled and divided into grids; **E.** The illustration shows stent movement towards the end of the blood vessel, simulating stent delivery to the region of stenosis.

A male patient with coronary artery disease and a type 0.1.1 bifurcation lesion (Medina classification), presenting with 70% stenosis in MV and 87% in the Side Branch (SB), was retrospectively selected. Coronary CTA DICOM data were imported into Mimics for segmentation based on grayscale differences between lumen and surrounding tissues, followed by smoothing in Wrap2017. The 3D reconstruction was performed according to Torii *et al.* [14]. Balloon, stent, and delivery system models were developed in SolidWorks and meshed using HyperMesh or ABAQUS. A sixfold balloon (C3D4R elements), an Endeavor Resolute^®^ drug-eluting stent, and a delivery guide rail aligned with the vessel centerline were used to replicate realistic deployment mechanics. Written informed consent was obtained from the patient (Fig. **1**).

The inner diameters of the Proximal Main Vessel (PMV), distal MV, and SB were 3.18 mm, 2.47 mm, and 2.09 mm, respectively. The vessel wall was meshed in HyperMesh using second-order hexahedral elements (C3D8R), while the stent was meshed with tetrahedral elements (C3D4R), ensuring a balance between accuracy and computational efficiency. The final model included 60,532 elements and 49,750 nodes. Additionally, to ensure the reliability of the results, mesh independence was confirmed.

### 2.3 Assignment of Material Properties.

The isotropic superelastic constitutive relationship of the material properties of the coronary artery wall was constructed based on the data obtained by Holzapfel *et al.* [15]. The energy function can be expressed by the formula:


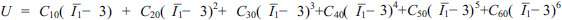


Where *I*_1_ represents the first invariant of the Cauchy-Green tensor, and *C*_10_ to *C*_60_ are material coefficients derived from experimental vessel data. This formulation accounts for the nonlinear mechanical behavior of arterial walls under stent-induced deformation (Table **1**) [15]. The blood vessel density was 1.12×10^-3^ g/mm^3^.

The material properties of the balloon were considered, with the balloon membrane thickness set to 0.025 mm. The balloon material was modeled as isotropic, with a Young’s modulus of 1.455 GPa, a Poisson’s ratio of 0.3, and a density of 1.256×10^-3^ g/mm^3^ based on the findings reported by Chiastra *et al.* [16]. The scaffold material, Cobalt Chromium alloy (CoCr)-commonly used in modern drug-eluting stents-was allocated with an elastic modulus of 233 GPa and a Poisson’s ratio of 0.35, as reported by Poncin *et al.* [17]. The yield strength was 414 MPa, the fracture stress was 933 MPa, and the fracture strain was 0.448.

### 2.4 Boundary Conditions

According to the commonly used pressure of stent balloon release in clinical interventional procedures, after the balloon was fully expanded, the pressure applied on the inner surface of the balloon was reduced to -0.2 MPa. Simultaneously, a negligible viscous resistance was applied to the surface of the blood vessel wall to allow the entire analysis process to reach a quasi-static equilibrium. No boundary conditions were imposed on the support. Explicit dynamic and quasi-static analyses were employed to simulate stent implantation and post-expansion.

### 2.5 Quantification of Clinical Indicators

This paper focuses on stent malapposition, the steel beam obstruction of the SB opening, the dilatation of a stenotic site, and vascular wall damage, all of which are related to adverse events, such as in-stent restenosis and thrombosis. The malapposition node considered in this study referred to a distance to the inner surface of the blood vessel of ≥ 260 μm. The stent malapposition was quantified as the percentage of stent attachment failure. The minimum lumen area of the stent segment was used to quantify the dilation of a vascular stenosis. The blood vessel wall stress distribution was used to evaluate damage to the blood vessel wall.

### 2.6 Clinical Validation

To validate the FEA results, a retrospective clinical study was conducted involving 50 patients diagnosed with coronary bifurcation lesions who underwent PCI at our institution. Patients were divided into two groups based on the double-stenting technique used: 25 patients received the Culotte stenting technique, and 25 underwent the Crush technique. Post-procedural IVUS imaging was performed in all cases to assess stent apposition and side-branch lumen patency. Furthermore, a follow-up was performed at 6 months to evaluate Target Lesion Revascularization (TLR).

### 2.7 Statistical Methods Used for Analysis.

Quantitative results from the retrospective clinical analysis were statistically compared between the two procedural groups. Continuous variables were expressed as mean ± standard deviation and analyzed using the independent samples Student’s t-test. Categorical variables were presented as frequencies or percentages and compared using the Chi-square test or Fisher’s exact test when appropriate. A p-value < 0.05 was considered statistically significant. All statistical analyses were performed using SPSS 21.0.

## 3 RESULTS

### 3.1 Expansion Process of the Two Double-Stent Procedures

The Cullotte procedure is shown in Fig. (**2A**). The process of the Crush procedure is illustrated in Fig. (**2B**).

### 3.2 Verification of Quasi-Static Nature of the Simulation Process

Explicit dynamic and quasi-static analyses were employed to simulate stent deployment. As shown in Fig. (**3**), the ratio of kinetic to internal energy remained below 5% after a brief initial fluctuation, indicating numerical stability. The convergence of external work and internal energy confirmed that the simulation could be considered quasi-static.

### 3.3 Effect on Vascular Stenosis

**Fig. (4):**
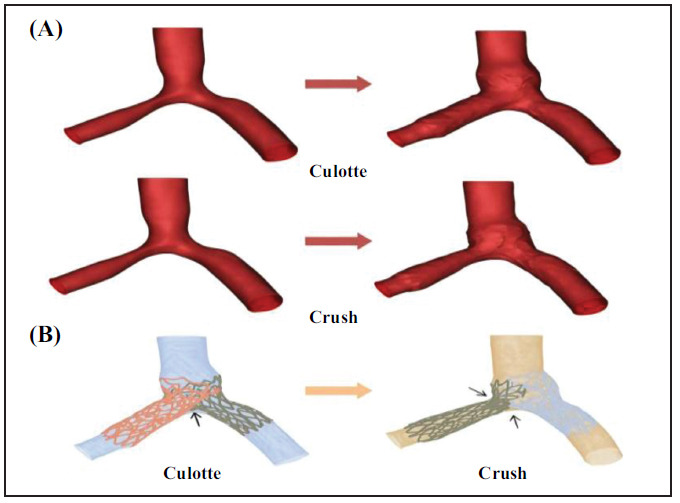
Comparison of beneficial effects and stent malapposition between the Culotte and Crush procedures on vascular stenosis. **A**. Comparison of the beneficial effects of the two procedures on vascular stenosis. The luminal areas of the MV and SB stent increased after both procedures, but to a greater extent after the Culotte procedure compared to the Crush method in both segments. **B**. Stent malapposition (indicated by arrows) observed after the Culotte and the Crush procedures.

Following Culotte stenting, the minimum lumen areas of SB and MV were 2.83 mm^2^ and 3.79 mm^2^, respectively. In comparison, the Crush technique yielded 1.76 mm^2^ (SB) and 3.62 mm^2^ (MV), indicating a superior lumen expansion with the Culotte method (Fig. **4A**). Stent malapposition occurred in 0.57% of nodes in the Culotte group and 1.26% in the Crush group. Malapposition was predominantly located near the SB opening in Culotte cases, and at the proximal edge in Crush cases (Fig. **4B**).

### 3.4 SB Opening Bracket Blocked and Vascular Wall Stress

**Fig. (5):**
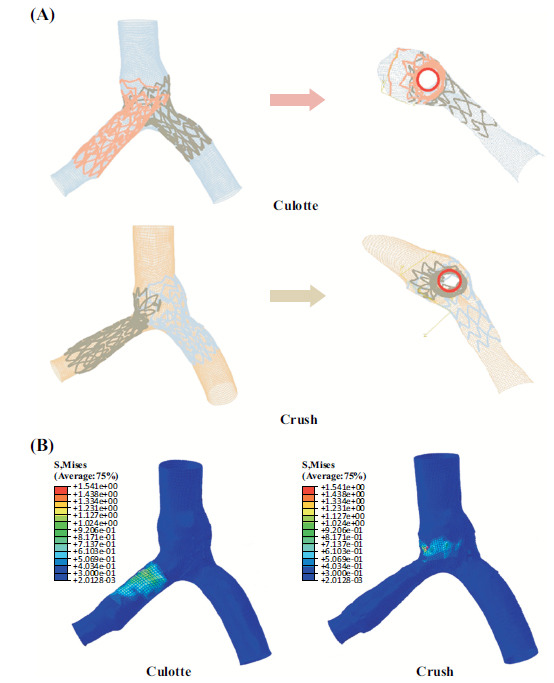
Comparison of the effects of two modes of open stent narrowing of the SB, and stress distribution on the blood vessel wall. **A**. Comparison of effects of the two stenting techniques on SB narrowing. The upper left panel indicates the effect of the Culotte technique, while the lower left panel shows the effect of the Crush technique. The right panels show the SB lumen (red circles) after the two methods. **B**. Stress distribution on the blood vessel wall after the two double stent procedures. The green hatched area indicates the main area of wall stress.

Stent obstruction at the SB opening was assessed in perpendicular cross-sections (Fig. **5A**, red circles). The Culotte technique showed 0.00% obstruction, while the Crush technique resulted in 3.76% obstruction.

Stress distribution on the vessel wall was visualized using the ABAQUS module. In the Culotte group, stress was primarily concentrated in the SB, whereas in the Crush group, stress was focused at the PMV, where three stent layers overlapped (Fig. **5B**).

### 3.5 Cross-Validation with IVUS

The simulation-predicted malapposition areas were compared with IVUS measurements, demonstrating a high degree of agreement, as shown in Fig. (**6**). Post-procedural IVUS assessments revealed that Culotte stenting was associated with a significantly lower rate of stent malapposition (3.2% *vs*. 8.6%, *p* = 0.027) and a higher minimal lumen area in the side branch (2.84 ± 0.51 mm^2^ *vs*. 2.11 ± 0.64 mm^2^, *p* = 0.013). Furthermore, follow-up at 6 months showed that the incidence of TLR was significantly lower in the Culotte group (12.0%) compared to the Crush group (28.0%, *p* = 0.042).

## 4 DISCUSSION

The optimal strategy for double-stenting in complex coronary bifurcations remains a topic of debate in contemporary interventional cardiology. The structural morphology of coronary stents and the local hemodynamic environment after stent deployment are the key determinants of technical success and clinical outcomes [18]. FEA represents a novel approach for studying and comparing two currently used stent techniques. However, most previous numerical simulation studies have focused on the optimization of stent design, including stent drug dose [19], shape [20], and construction material [21], among others. In practice, most clinicians focus on selecting the optimal interventional method for individual patients. Clinically, stent adhesion can be measured using OCT/IVUS, but this may increase procedure cost and duration. In view of the ongoing clinical need, this simple numerical simulation allows for post-procedure visual analysis of the stent and blood vessels, and can also be used to analyze the advantages and disadvantages of the two procedures in the same lesion model, potentially guiding clinical decision-making.

The study found that the double stent morphologies were more closely matched with the geometry of the bifurcation vessels after the Culotte procedure, which improved, but did not completely eliminate poor stent attachment at the proximal end of the stent and the bifurcation ridge. This procedure produced a “metal artificial vessel bifurcation”. After the release of the MV stent, the SB stent was squeezed, collapsing/deforming the two-layer metal stent at the opening of the SB, leading to poor adhesion of the stent at the bifurcation ridge and displacing the stent at the SB vessel wall, thereby reducing adhesion. In double-stent procedures, this stretching effect, which tends to displace the bifurcated spine, may explain the clinical need for SB rewiring. We also found that the branch balloon may not achieve full expansion under the normal ‘safe’ inflation pressure due to the large branch angle, resulting in reduced SB dilation with the Crush compared to the Culotte technique. It is recommended that clinicians regularly check the effectiveness of stent extrusion and carefully select the appropriate balloon size.

Previously, it was reported that the choice of double-stent procedure was the most important factor affecting the rate of restenosis and the SB lumen area [22]. These results demonstrated that the degree of opening was less after Crush stenting than after the Culotte method. This may be because the SB balloon of the Crush method compressed the stent at the SB opening on the side opposite to the bifurcation ridge, as the redundant stent ring is squeezed around the side of the MV and the SB opening. Following the release of the MB stent, the distal side of the SB stent opening may still be squeezed by the MV, leading to repeat collapse. Consequently, a small amount of the stent remains at the SB opening, reducing the aperture. In contrast to the Crush method, the Culotte technique showed no stent obstruction at the SB after full expansion of the MB stent, opening of the mesh, and insertion of the SB stent. In addition, the stress distribution map findings suggest that the stress following the Culotte procedure was mainly concentrated in the SB vessel. While the Crush method led to stress concentrated in the three-layer stent overlap at the proximal end of the MV. Compared to the Cullotte procedure, the stress after the Crush technique was increased locally, potentially injuring the proximal end of the MV through excessive expansion, which may also account for the increased incidence of MACE reported after the Crush procedure [23]. Intravascular imaging plays a pivotal role in optimizing bifurcation PCI by guiding stent sizing, positioning, and evaluating post-procedural results. Recent evidence has emphasized the importance of imaging-based strategies in improving clinical outcomes for bifurcation lesions [24]. This study incorporated IVUS to validate the simulation findings, highlighting the potential of integrating computational models with imaging tools in procedural planning.

Overall, from the perspective of stent deformation and mechanics, the Culotte procedure showed a more favorable profile than the Crush procedure, which is consistent with the clinical observations of Kahraman *et al.* [25]. However, the interventional strategy mainly depends on the patient characteristics and clinicians’ decisions, with procedural success also dependent on the experience of the operator. Additionally, patient prognosis may also be impacted by duration of the procedure, fluoroscopy time, and contrast agent load involved in the different double-stent strategies [26, 27].

The FEA results in this study demonstrated that the Culotte technique resulted in better stent apposition, lower side-branch obstruction, and reduced vessel wall stress compared to the Crush technique. The results of this FEA are intended to provide biomechanical insights into how different stenting techniques may affect vessel morphology and stress distribution under standardized conditions. FEA, while currently limited by computational complexity and simplifications in material and boundary assumptions, holds promise as a complementary tool to assist clinicians in understanding procedural mechanics and optimizing interventional strategies. Future studies should focus on integrating FEA with preprocedural imaging techniques (*e.g.*, OCT/IVUS) and real-time procedural planning to further personalize PCI strategies and improve patient outcomes. Additionally, future studies could extend the current modeling platform to simulate the deployment and mechanical behavior of newer-generation ultrathin-strut stents and bioresorbable polymer-based scaffolds. These devices may offer distinct advantages in bifurcation lesions due to their improved flexibility and reduced chronic inflammation risk. Investigating their performance through FEA could provide valuable insights into device selection for complex anatomies.

## 5 LIMITATIONS

Although this study provides valuable biomechanical insights into double-stent implantation strategies, several limitations should be acknowledged. First, the EFA was based on a single patient-specific model, which may not capture the full range of anatomical variations encountered in clinical practice. Second, the material properties of the vessel and stent were assumed to be homogeneous and isotropic, which may not fully reflect the complex behavior of biological tissues. Third, hemodynamic factors, such as blood flow dynamics and shear stress, were not incorporated into the simulations. Finally, while the findings were validated against IVUS data, larger-scale clinical validation is required to confirm their generalizability. Future studies should include more patient-specific geometries, integrate fluid-structure interaction, and explore clinical outcome correlations.

## CONCLUSION

This study evaluated the biomechanical performance of Culotte and Crush stenting techniques for coronary bifurcation lesions using finite element analysis. The Culotte approach showed favorable mechanical characteristics, suggesting it may be more suitable in specific anatomical settings. These findings highlight the value of biomechanical simulation in guiding stenting strategy selection. Future research is warranted to validate these insights in broader clinical contexts.

## Figures and Tables

**Fig. (2):**
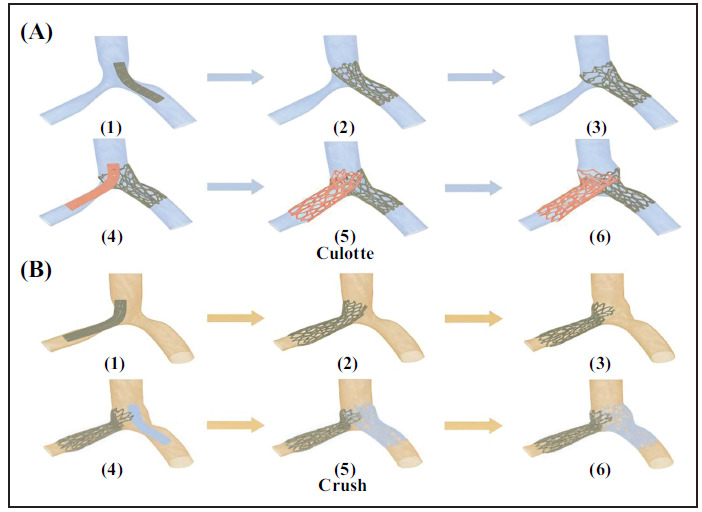
Double-stenting according to the culotte and crush procedures. **A.** Double-stenting according to the Culotte procedure. (1) An appropriate sized stent (green) is inserted in the MV, covering the stenosis, (2) The balloon is expanded and (3) withdrawn, (4) An appropriate sized stent (orange) is inserted in the SB through the mesh of the MV stent, and (5) overlapping of the two stents creates the typical ‘waist sign’ of the Culotte procedure, and (6) final kissing balloon dilatation of the PMV is performed. **B.** Double-stenting according to the Crush procedure. (1) An appropriately sized stent (green) is inserted in the SB, (2) The stent is expanded, (3) The balloon that expanded the SB stent is withdrawn, pressing the SB stent against the PMV. (4) A second stent (gray) was placed in the MV, (5) the second stent is expanded, forming a structure with three layers of stent on one side and one layer of stent on the other side in the PMV. (6) Finally, kissing balloon dilatation of the PMV was performed, and the balloon was withdrawn.

**Fig. (3):**
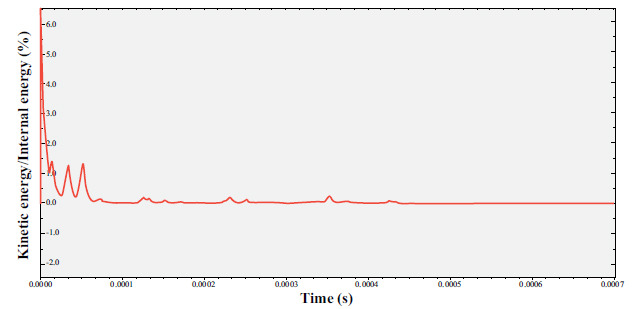
Ratio of kinetic to internal energy over time (in seconds) during the simulation process.

**Fig. (6):**
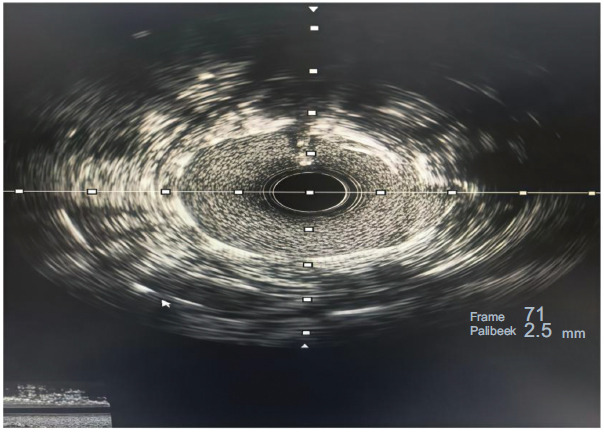
IVUS Validation of computational stent deployment model.

**Table 1:** Material properties of the blood vessel wall.

**-**	***C*_10_**	***C*_20_**	***C*_30_**	***C*_40_**	***C*_50_**	***C*_60_**
Vessel wall	6.52E-03	4.89E-02	9.26E-03	7.60E-01	-4.30E-01	8.69E-02

## Data Availability

All data generated or analyzed during this study are included in this published article.

## LIST OF ABBREVIATIONS

CBLS: Coronary Bifurcation Lesions
CTA: Computed Tomography Angiography
DICOM: Digital Imaging and Communications in Medicine
FEA: Finite Element Analysis
IVUS: Intravascular Ultrasound
MACE: Major Adverse Cardiovascular Events
MV: Main Vessel
PCI: Percutaneous Coronary Intervention
PMV: Proximal Main Vessel
POT: Proximal Optimization Technique
SB: Side Branch
TLR: Target Lesion Revascularization

## STANDARDS OF REPORTING

STROBE guidelines were followed.
